# Early diagnosis of femoral neck stress fractures may decrease incidence of bilateral progression and surgical interventions: A case report and literature review

**DOI:** 10.1016/j.ijscr.2018.10.050

**Published:** 2018-10-31

**Authors:** Kruten M. Patel, Brian A. Handal, William K. Payne

**Affiliations:** a Doctors Medical Center, Orthopedic Surgery Department, Graduate Medical Education, 1400 Florida Avenue Suite 200, Modesto, CA, 95350, United States; b Grandview Medical Center, Orthopedic Surgery Department, Graduate Medical Education, 405 W. Grand Ave, Dayton, OH, 45405, United States; c Department of Orthopedic Surgery, Franciscan St. James Hospital, 20201 South Crawford Avenue, Olympia Fields, IL, 60461, United States

**Keywords:** Osteoporosis, Bilateral femoral neck fractures, Stress fractures, Vitamin D deficiency, Case report

## Abstract

**Introduction:**

Early intervention in femoral neck stress fractures (FNSFs) can be self- limiting, but they have an insidious presentation. High index of suspicion for an occult fracture is necessary to avoid bilateral progression and/or operative interventions.

**Case presentation:**

A 83-year-old female, non-athlete, presents with multiple comorbidities and progressive bilateral FNSFs. Initially the patient presented with groin pain and radiographs demonstrated a non-displaced compression type fracture of the right femoral neck without any inciting events. Subsequently, during a post-operative visit of her right hip, a work-up for new onset contralateral groin pain revealed a non-displaced tension type fracture on the left femoral neck. Both FNSFs were treated with percutaneous cannulated screw fixation.

**Discussion:**

Vitamin D deficiency, poor nutrition, and osteoporosis have been associated with developing stress fractures. This presents an interesting question of whether these frequently referenced risk factors play an interrelated role. Treatment algorithms are controversial, but have been successful in preventing the progression of occult stress fractures. Yet, identification of FNSFs represents a major challenge in diagnosis for clinicians.

**Conclusion:**

This case report documents an uncommon fracture pattern in the elderly population. With an aging population, it is pertinent to avoid missed opportunities for prompt diagnosis and implementation of noninvasive methods of treatment. Therefore, paying attention to the risk factors with a high index of suspicion would be ideal.

## Highlights

• Stress Fractures in elderly patients are rarely discussed.
• Vitamin D deficiency, osteoporosis, and weight are important comorbidities to consider for these stress fractures.
• Have a high index of suspicion when multiple risk factors are observed in a patient.
• Patients can be treated non-operatively or with minimally invasive procedures if the issue is identified early.

## 1 Introduction

The diagnosis of stress fractures became clearly defined in the 1960s through case reports described by Devas and Ernst [1,2]. Since that time there have been several publications describing the epidemiology, diagnosis, and treatment of these insidious fracture patterns. To understand stress fractures, we must develop a delineation between the pathophysiology of fatigue and insufficiency fractures. Fatigue types occur when excess stress is put on a bone with normal strength. Insufficiency types occur when normal stress is put on abnormal bone [3]. Interestingly, many risk factors in the elderly for insufficiency types have been reported including osteoporosis, osteomalacia, long-term corticosteroid use, bisphosphonate use, and vitamin D deficiency [[4], [5], [6]]. Our patient mirrored these risk factors with vitamin D deficiency, osteoporosis, and bisphosphonate use. A prospective study by Fullerton further categorized FNSFs as compression and tension types [3]. Compression type FNSFs were seen to occur inferior-medial on the femoral neck while tension type is superior-lateral. This is important because it can affect the treatment protocol. The key to treatment is early diagnosis to decrease the risk of further progression to displacement and collapse. Compression type fractures of the femoral neck are mechanically more stable, so a trial of non-operative treatment with frequent radiographic follow-up may be considered [7,8]. Tension types of stress fractures are potentially unstable and require screw fixation or prosthetic replacement depending on the fracture characteristics [8,1]. The work has been reported in line with the SCARE criteria [9].

Since the initial reports of stress fractures, there has been added emphasis on FNSFs. Yet, the emphasis has been focused mainly on active young individuals. Our report highlights the most important comorbidities and stresses the importance of prevention or early diagnosis for the elderly. To our knowledge, there has been no report of a case where an elderly patient suffered bilateral FNSFs. The following case describes the development of bilateral FNSFs within a 6- month time frame.

## 2 Case report

**Fig. 1:**
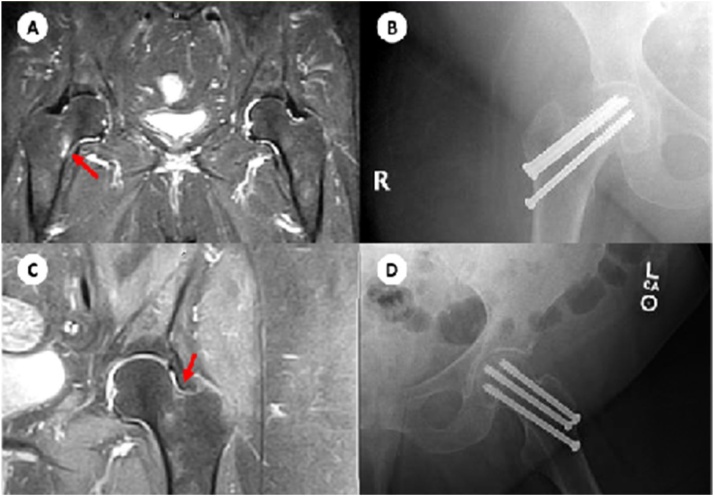
(A) MR study of the pelvis which demonstrates a right femoral neck stress fracture on the compression side type that involves >50% of the cortical bone. (B) Radiograph of the right hip demonstrating postoperative changes status-post closed reduction percutaneous pinning of the femoral neck using three cannulated screws. (C) MR study of the left hip which demonstrates a left femoral neck stress fracture on the tension side that is unicortical. (D) Radiograph of the left hip demonstrating postoperative changes status-post closed reduction percutaneous pinning of the femoral neck using three cannulated screws.

An 83-year-old female presented on two separate occasions with spontaneous groin and hip pain. Using MRI, the patient was diagnosed with an initial right compression type FNSF and subsequently with a left tension type FNSF. Of worthy mention, the patient has multiple comorbidities including: morbid obesity, hypertension, type 2 diabetes mellitus, osteoarthritis, osteoporosis, and vitamin D deficiency. Her Vitamin D level was 15 ng/mol. Pertinent review of medications includes: Ergocalciferol 50,000 units and Alendronate 70 mg. At the initial encounter, the patient presented for a month- long duration of hip pain that radiates to the groin and aggravated by minimal activity. On evaluation, she complained of anterior groin pain with all directions of motion of the hip and no other pertinent physical exam findings. MRI of the lower right extremity demonstrated high signal intensity edema in the bone marrow at the inferior medial aspect of the right femoral neck; findings that are consistent with a compression stress reaction (Fig. 1). After thorough consideration of the patient’s past medical history and review of the risks/benefits of a surgical procedure, the decision was made to treat her FNSF with percutaneous pinning with cannulated screw internal fixation. Postoperatively, the patient was treated weight bearing as tolerated with home physical therapy three to four times a week and followed closely in one month intervals. Three months later, the patient presented for a follow-up status-post internal cannulated screw fixation of her right hip with new onset pain on the contralateral side and inability to ambulate. At that time her Vitamin D level was 25 ng/ml. On evaluation, she complained of groin pain that is exacerbated by leg roll and present for a 3- week duration. Ranging of the hip joint revealed exquisite pain restricted to internal and external rotation flexed at 90°. MRI of the lower left extremity demonstrated high signal intensity edema in the bone marrow at the superior-lateral aspect of the left femoral neck (Fig. 1). These findings are consistent with a tension stress reaction, as opposed to a typical inferior medial aspect stress fracture. Risks and benefits of surgery for the left hip were discussed with the patient and the patient opted to receive cannulated screw fixation. At her 6 week follow-up, the patient had improved ambulation and decrease in pain.

## 3 Discussion

Stress fractures are most commonly seen due to overuse. These injuries are regularly misdiagnosed with upto 75% of femoral neck stress fractures being missed [4]. Generally, there is a history of overuse and presents as an insidious onset of pain which improves with cessation of activity. Physical exams are usually benign, but may have tenderness to deep palpation or groin pain with internal and/or external rotation of the hip [10]. Therefore, it is important to pay attention to the radiographic signs since visualization of the actual fracture line may be difficult. Initially, x-rays can show a periosteal thickening demonstrating a periosteal reaction [11]. Other signs include periosteal sclerosis, cortical changes and lateral callus formation [11]. However, the gold standard for diagnosis is a MRI with T1-weighted images showing a linear hypointense fracture line [12]. The pathognomonic description is a high-intensity signal of edema on the superior-lateral or inferior-medial aspects of the femoral neck. An MRI is crucial in achieving an early diagnosis.

Many comorbidities are associated with stress fractures. Most important risk factor to consider is Vitamin D levels. It has been highlighted in multiple accounts with a study specifically mentioning it seen in 83.8% of their elderly stress fractures [13]. Also, Vitamin D deficiency is greater in older individuals due to poor nutrition, insufficient sunlight exposure, and chronic kidney disease. The decreased mineralization softens the bone making it susceptible to fractures. Also, osteoporosis is another issue that frequently presents with insufficiency fractures. Osteoporosis affects over 10 million people in the US [14]. Once women go through menopause, the bone resorption occurs at a higher rate than build up due to lack of estrogen. Thus, elderly females usually over the age of 65 suffer from this disease. The reported prevalence was as high as 50% in the literature [15]. There is limited information on higher BMI affecting insufficiency fractures with only one article discussing how a BMI over 25 was discovered in 40.4% of their patients [13]. Yet, we feel it is an important modifiable comorbidity due to the fact that an increase in weight puts excess stress on an already defective weight bearing bone. With an aging population and an increased prevalence of osteoporosis, we also see increased usage of bisphosphonate therapy. The relationship between bisphosphonate therapy and a risk for stress fractures in the elderly are well documented in the literature. It has been shown that the increased fracture risk associated with the use of these antiresorptive meds reach upwards of 30% after 5 years of use [10]. Our patient only recently started this therapy, shortly after the right FNSF. Thus, it is highly unlikely it played a role in the development of her FNSFs. Table 1 highlights the occurrence of risk factors from 2 case series of a total of 143 elderly patients that suffered stress fractures [13,15]. Addressing issues on this table is important when prevention is considered as well as in the setting of early diagnosis.

**Table 1:** Incidence of Risk Factors in Elderly Stress Fractures.

Risk Factor	Incidence
Gender (F/M)	122/142 (85.9%)
Vitamin D Deficiency	60/72 (83.8%)
BMI	33/82 (40.2%)
Osteoporosis	50/141 (35.5 %)
PPI use	18/80 (22.5%)
Cortisone Therapy	22/140 (15.7%)

**Fig. 2:**
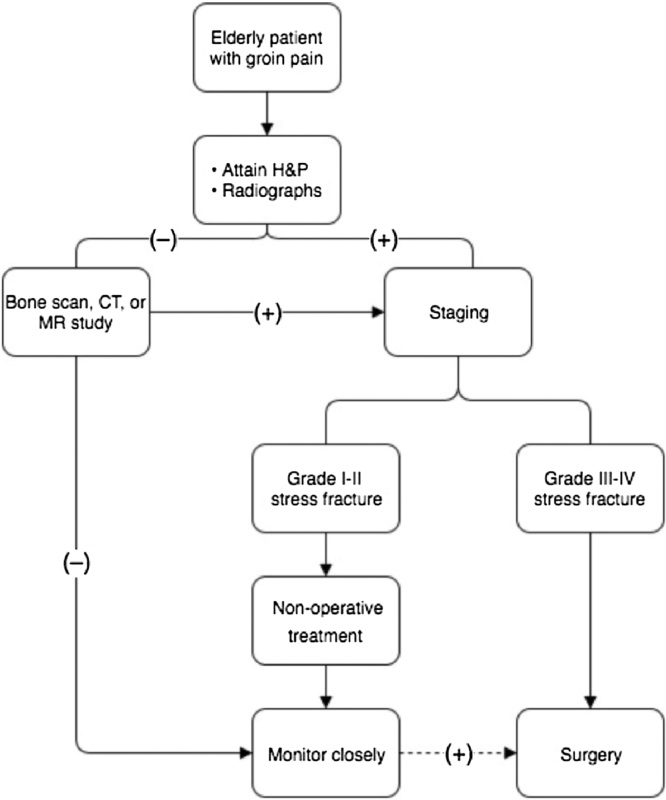
Algorithm for evaluation and treatment of femoral neck stress fractures in the elderly.

Treatment of femoral neck stress fractures is still a diagnostic challenge for surgeons. We have formulated a treatment algorithm geared toward femoral neck stress fractures in elderly patients (Fig. 2). Additionally, it is helpful to utilize a proposed classification system for grading stress fractures with the purpose of guiding treatment as Boden et al did.16 Grade 1–2 FNSFs are typically unicortical with <50% of bone involved. Grade 3 FNSFs involve >50% of the bone and grade 4 FNSFs are bicortical. As described earlier, the first and most challenging step in treatment is identification. Most cases can be diagnosed with a thorough history, physical examination, and radiographs [16]. In the elderly population it is important to keep a high index of suspicion due to a high-risk for progression to complete fracture. Frequently, the end goal of treatment is surgical, especially in any tension type FNSFs. The exception is the unicortical compression stress fracture that is typically a grade 1–2 FNSF where a trial of non-operative treatment may be considered. Boden et al recommend non-weight bearing in low-demand athlete with non-displaced fractures [16]. However, in the elderly maintain a non-weight bearing status is unreasonable and difficult; therefore, we propose a more aggressive surgical recommendation for this population.

## 4 Conclusion

Bilateral femoral neck stress fractures in the elderly are uncommon. Research is limited on the topic with vitamin D deficiency as the major determinant. This case report aims to shift attention to the importance of early recognition and more aggressive treatment, which will prevent the progression to a displaced fracture as well as initial fracture development. We suggest clinicians to go over Table 1 and keep a high-index of suspicion as signs and symptoms of stress fractures are observed. Having a high-index of suspicion will lead to prevention, non-operative treatment, or minimally invasive surgical options.

## Conflicts of interest

No conflict of interest.

## Funding

No funding.

## Ethical approval

No approval needed since our ethical approval was exempted by our institution since written consent was obtained.

## Consent

Written informed consent was obtained from the patient for publication of this case report and accompanying images. A copy of the written consent is available for review by the Editor-in-Chief of this journal on request.

## Author contribution

Kruten Patel – Researched and wrote report.

Brian Handal – Researched and wrote the report.

William Payne – Reviewed and helped make revisions in the paper.

## Registration of research studies

Case report not a clinical study.

## Guarantor

Kruten Patel, Brian Handal, and William K Payne.

## Provenance and peer review

Not commissioned, externally peer reviewed.
